# Cataphoresis in Dental Practice

**Published:** 1897-04

**Authors:** E. Grosheintz, B. J. Cigrand

**Affiliations:** Basel.


ARTICLE II.

### CATAPHORESIS IN DENTAL PRACTICE.

BY DR. E. GROSHEINTZ, BASEL.

The word cataphoresis (from the Greek, meaning to carry away or wash along) suggests to our mind a picture of a river carrying with it floating material. The physicist defines cataphoresis as that property or power of electricity to conduct or exchange from the positive to the negative pole a fluid through a porous body. If we conduct a constant current through a tissue or body we can experience the fluid passing from the positive pole (anode) in the direction of the negative pole (kathode); and this phenomenon we term cataphoresis.

The physicians understand by the word the forcing of medicine into the body by means of a current of electricity. Prof. W. J. Morton has employed a very satisfactory expression for this process in this phrase: "Electric—medicamental—diffusion." Cataphoresis is a purely mechanical process and in this particular differs from the chemical process accompanying electrolysis. And it is possible for electrolysis and cataphoresis to act in conjunction, and it is difficult, or rather impossible, to determine just how great a functicn each exercise.

The earliest instance in which catephoresis was employed in the practice of medicine seems to have been in France. As early as 1835 the introduction of tinct. of iodin was practiced, and for upwards of twenty years the French specialists recommended it as productive of good results in the treatment of several internal disorders. The learned scientists of the several continents have from time to time studiously occupied themselves in the investigation of cataphoretic experiments without establishing for it a specific purpose in general practice. Only recently the Americans have, under the great electrician, Edison, taken up the oft neglected study of electricity, and now promise many positive results.

Dr. William J. Morton, professor of nervous diseases and electro-therapeutics in New York, is the son of the renowned Dr. Morton, the discoverer of etherial anesthesia; and the subject of cataphoresis in receiving the special research of the young Dr. Morton. He is studying it relative to its worth in the science of dental surgery. We are indebted to him for having substituted cataphoretic anesthesia in the place of cocain; and his success with guaiacol-cocain is deserving of considerable notice. On May 12, 1892, Dr. Westlake, of Elizabeth, New York, in an essay before the State Dental Society said: "I am convinced that the problem of obtunding sensitive dentine will be solved through the agency of the electro-medication or cataphoresis." I have experimented considerably with electricity, relative to discovering the latent properties which might prove of service to our profession, and I am certain of valuable aid coming from its further employment. I use it in bleaching teeth; in disinfecting root-canals; in painless extirpation of the pulp; in extracting teeth; in the treatment of pericementitis, and in alveolar pyorrhea.—Translated by Dr. B. J. Cigrand from *Schweizerische Vierteljahrschrift fuer Zahnheilkunde.*

